# Quantitative MRI analysis in children with multiple sclerosis: a multicenter feasibility pilot study

**DOI:** 10.1186/1471-2377-13-173

**Published:** 2013-11-13

**Authors:** Tanuja Chitnis, Charles R Guttmann, Alexander Zaitsev, Alexander Musallam, Bianca Weinstock-Guttmann, Ann Yeh, Moses Rodriguez, Jayne Ness, Mark P Gorman, Brian C Healy, Nancy Kuntz, Dorothee Chabas, Jonathan B Strober, Emmanuelle Waubant, Lauren Krupp, Daniel Pelletier, Bradley Erickson, Niels Bergsland, Robert Zivadinov

**Affiliations:** 1 Partners Pediatric Multiple Sclerosis Center, Massachusetts General Hospital for Children, 55 Fruit St, ACC 708, 02114 Boston, MA, USA; 2 Center for Neurological Imaging, Brigham and Women’s Hospital, Boston, MA, USA; 3 The Pediatric MS Center at the Jacobs Neurological Institute, SUNY-Buffalo, Buffalo NY, USA; 4 Department of Neurology and Immunology, Rochester, MN, USA; 5 Department of Radiology, Mayo Clinic, Rochester, MN, USA; 6 Department of Pediatric Neurology, University of Alabama, Birmingham, AL, USA; 7 Biostatistics Center, Massachusetts General Hospital, Boston, MA, USA; 8 Department of Neurology, University of California, San Francisco, USA; 9 Department of Neurology, SUNY-Stonybrook, Stonybrook, NY, USA; 10 Buffalo Neuroimaging Analysis Center, Jacobs Neurological Institute, SUNY-Buffalo, Buffalo, USA; 11 Department of Pediatrics Mayo Clinic, Rochester, MN, USA

## Abstract

**Background:**

Pediatric multiple sclerosis (MS) is a rare disorder with significant consequences. Quantitative MRI measurements may provide significant insights, however multicenter collaborative studies are needed given the small numbers of subjects. The goal of this study is to demonstrate feasibility and evaluate lesion volume (LV) characteristics in a multicenter cohort of children with MS.

**Methods:**

A common MRI-scanning guideline was implemented at six member sites of the U.S. Network of Pediatric MS Centers of Excellence. We included in this study the first ten scans performed at each site on patients meeting the following inclusion criteria: pediatric RRMS within 3 years of disease onset, examination within 1 month of MRI and no steroids 1 month prior to MRI. We quantified T2 number, T2-LV and individual lesion size in a total of 53 MRIs passing quality control procedures and assessed gadolinium-enhancing lesion number and LV in 55 scans. We studied MRI measures according to demographic features including age, race, ethnicity and disability scores, controlling for disease duration and treatment duration using negative binomial regression and linear regression.

**Results:**

The mean number of T2 lesions was 24.30 ± 19.68 (range:1–113) and mean gadolinium-enhancing lesion count was 1.85 ± 5.84, (range:0–32). Individual lesion size ranged from 14.31 to 55750.60 mm^3^. Non-white subjects had higher T2–LV (unadjusted p_T2-LV_ = 0.028; adjusted p_T2-LV_ = 0.044), and maximal individual T2-LV (unadjusted p_Max_ = 0.007; adjusted p_Max_ = 0.011) than white patients. We also found a trend toward larger mean lesion size in males than females (p = 0.07).

**Conclusion:**

Assessment of MRI lesion LV characteristics is feasible in a multicenter cohort of children with MS.

## Background

Multiple sclerosis (MS) is an increasingly recognized disorder in children and adolescents. The onset of MS prior to the age of 18 occurs in 3-5% of the total MS population [1-3]. Children and adolescents with MS have higher relapse rates than adults with the disease [4], suggesting inflammation as a prominent feature. Children also demonstrate considerable cognitive disability early in the disease course [5-7] but relatively less locomotor disability than adults with a similar disease duration [8].

MRI features of pediatric MS may provide further insight into these clinical observations. Studies have suggested that younger children with MS present with atypical MRI features [9], and brain T2 lesion load appears relatively higher at disease presentation in pediatric-onset patients than adult-onset MS [10]. Others have suggested gender differences in lesion location in children with clinically isolated syndromes [11].

The goal of this cross-sectional multicenter pilot study was to evaluate MRI burden of disease using quantitative measures in a cohort of children and adolescents with MS early in their disease course. In addition, we studied MRI measures according to demographic features including age, race and ethnicity, and disability scores.

## Methods

### Patients

MS patients were seen at one of the six U.S. Network of Pediatric MS Centers of Excellence located in San Francisco, CA (UCSF), Rochester, MN (Mayo), Birmingham, AL (UAB), Buffalo, NY (SUNY Buffalo), Stony Brook, NY (SUNY Stony Brook), and Boston, MA (MGH). In this pilot study, we obtained MRI scans from the first 10 subjects at each site (60 subjects total) who met the following inclusion criteria: 1) Diagnosis of RRMS and onset under 18 years using the International Pediatric MS Study Group diagnostic criteria [12]; 2) MRI performed using our standardized Network Pediatric MS MRI guidelines; 3) MRI performed less than 3 years from first symptom onset; 4) Clinical examination performed within 1 month of MRI (no acute relapse in between MRI and examination); and 5) No steroids administered 30 days prior to MRI scans. We collected MRI scans from ten patients at each site. Demographic data, including age at disease onset, EDSS, self-identified race and ethnicity and treatment history, were collected using a standardized database and are presented in Table 1. This study was conducted in compliance to the Helsinki Declaration. We obtained IRB approval for this study at all sites from the following ethics boards: Partners Human Research Committee (MGH), Health Sciences Institutional Review Board (SUNY-Buffalo), Stonybrook University IRB (SUNY-Stonybrook), University of Alabama IRB (UAB), UCSF Human Research Protection Program (UCSF), Mayo Clinic IRB (Mayo Clinic). Signed informed consent from a parent or guardian for children under the age of 18 was required by the IRB boards at all sites except MGH.

**Table 1:** Baseline demographic and MRI features of subject cohort

**Characteristic**	
Number of subjects (N)	57
Gender [N (%) female]	38 (66.7%)
Race [N (%) self-reported white]	40 (70.2%)
Ethnicity [N (%) self-reported Hispanic/Latino]	14 (24.5%)
Age [years, mean+/−SD, (range)]	14.78 +/− 3.12 (5, 18)
Disease duration [days, mean+/−SD]	564.04 +/− 440.69
Disease modifying therapy (DMT) [N (%)]	38 (66.7%)
Time on DMT [days, mean +/− SD, (range)]*	206.57 +/− 207.76 (0, 702)
EDSS [mean +/− SD, (range)]**	1.1 +/− 1.0 (0, 4.0)
EDSS [median, (range)]**	1.0 (0.4.0)
T2 lesion count [mean +/− SD, (range)]^^^	24.30+/−19.68 (1,113)
T2 lesion total volume [mm^3^, mean +/− SD, (range)]^^^	10489.28+/− 12477.55 (55.37, 61472.37)
GD + lesion count [mean +/− SD, (range)]^†^	1.85 +/− 5.84, (0, 32)
GD + lesion volume [mm^3^, mean +/− SD, (range)]^††^	710+/− 1739, (60, 7536)

*Legend*: * = 3 subjects missing, ** = 1 subjects missing, ^^^=4 subjects missing, ^†^ = 2 subjects missing, ^††^ = among 19 subject with GD + lesions, EDSS = Expanded Disability Status Scale.

### MRI acquisition

Subjects underwent MRI at 1.5 T. Four sites used a 1.5 T General Electric (Milwaukee, WI, USA) Signa Excite HDx scanner. One site used Philips (Best, the Netherlands) Intera Gyroscan scanner, and another site used Siemens (Erlangen, Germany) Avanto scanner. The Pediatric MS Network protocol consisted of a standardized protocol. The protocol included a two-dimensional (2D) double-echo proton density (PD) and T2-weighted (T2W) spin-echo (SE) sequence [repetition time (TR) = 3200–3500 ms, first and second echo time (TE_1_/TE_2_) = 12-15/90-105 ms, FA = 90°, field of view (FOV) = 256 mm, phase FOV (pFOV) = 75%, acquisition matrix = 256×192 for an in-plane resolution of 1×1 mm, slice thickness = 3 mm, NEX = 1], a 2D Fluid Attenuated Inversion Recovery (FLAIR) sequence [TR = 8000–11000 ms, TE = 93–140 ms, inversion time (TI) = 2200–2250 ms, FA = 90°, FOV = 256 mm, pFOV = 75%, acquisition matrix = 256×192 for an in-plane resolution of 1×1 mm, slice thickness = 3 mm, NEX = 1], a 2D T1-weighted (T1-W) SE sequence post-contrast contrast agent injection (0.1 mmol/kg with 5 minutes of delay) [TR = 500–717 ms, TE = 20 ms, FA = 90°, FOV = 256 mm, pFOV = 75%, acquisition matrix = 256×192 for an in plane resolution of 1×1 mm, slice thickness = 3 mm, NEX = 1], and a sagittal three-dimensional (3D) T1W spoiled gradient recalled echo (SPGR) sequence.

### MRI analysis

#### Image distribution and quality control

We used an image-transfer and process- dispatching system based on service-oriented architecture (SOA). Data flow and process-coordinating webservices (WS) were deployed at the Center for Neurological Imaging, BWH, Boston, MA. A data-flow system with central quality control for series identification, protocol compliance, visual inspection was implemented at BWH. Lesion segmentation and volumetric analysis were performed at the Buffalo Neuroimaging Analysis Center, Buffalo, NY. Operators were blinded to patient clinical status.

#### Lesion numbers and volume

Calculation of the number, size and total volume of T2 lesions was performed on FLAIR images that were co-registered to PD/T2-WIs. The gadolinium-enhancing (GD+) lesion analysis was performed on SE-T1-WIs, whereas the 3D T1-SPGR images were used as a reference to confirm no presence of hyperintensities on corresponding pre-contrast scans. The analysis was based on a semi-automated tracing method using computer-displayed images, as previously described. [10,13] For each individual lesion, the total number of voxels contained within the lesion was calculated using a fully automated connected-components algorithm [14]. The size for each lesion was then obtained by multiplying the number of voxels circumscribed within the lesion by the volume of the voxel. We recorded total volume of lesions as well as individual lesion size for each subject.

#### Statistical methods

The main outcome measures for this study were the number of T2 lesions, number of GD + lesions, total T2 lesion volume (LV) and maximum lesion size for each subject. The potential predictors of these outcome variables were demographic (age, gender, race, and ethnicity) or clinical (EDSS). For all analyses, the lesion volumes were log-transformed to account for the right skew in the data. In each analysis, univariate and multivariate models that adjusted for disease duration and treatment duration at the time of the MRI scan were fit. The associations between the total lesion number and the potential predictors were assessed using negative binomial regression, and the association between the log-transformed total lesion volumes and the potential predictors were assessed using linear regression. In addition to these analyses, we also measured the size of each individual lesion in each subject (1288 T2 lesions in 53 subjects with MRIs that passed T2 sequence QC). To assess the effect of the predictors on the average lesion size, a repeated-measures model with a compound symmetry covariance matrix was used. This model accounted for the potential correlation of lesion sizes within a subject. Data analysis was performed utilizing the statistical software package, SAS Version 9.1.3 Service Pack 4, and a p-value of <0.05 was considered statistically significant in all analyses.

## Results

### Patients

**Figure 1:**
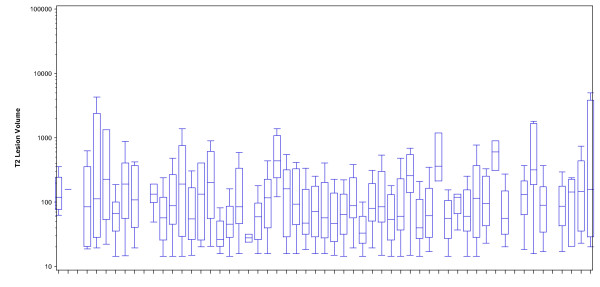
**Box-plot of volumes of T2 lesions within each subject (mm**^**3**^**): lesion size is plotted on a log-transformed scale.**

MRI scans from 57 subjects passed general quality control criteria and underwent quantitative MRI analysis. Baseline demographic and MRI characteristics of these subjects are summarized in Table 1. The demographic characteristics of the pediatric MS subjects were similar to other studies in the U.S. [3,4] in that the percentage of subjects self-identifying as non-white or Hispanic was higher than in typical adult cohorts. In addition, the mean disease duration since first symptom was less than two years so that the observed MRIs were performed soon after disease onset. The mean MRI measures are also listed in Table 1. Of the 57 available scans for MRI analysis, we calculated total T2-LV and T2 lesion numbers from 53 scans, since four scans did not pass quality control for T2 measures. Of these 53 subjects, all had at least one T2 lesion. Similarly, because of quality control, total Gd-LV and number was calculated from 55 scans, 19 (34.5%) of which had at least one Gd + lesion. The mean number of T2 lesions was 24.30 ± 19.68 (range:1–113), and mean gadolinium-enhancing lesion count was 1.85 ± 5.84, (range:0–32). Mean T2-LV was 10489.28 ± 12477.55, and mean gadolinium-enhancing LV was 710.35 ± 1739.87. Figure 1 depicts box-plots of individual T2 lesion size within each subject. Individual lesion size ranged from 14.31 to 55750.60 mm^3^. The mean (SD) of the difference between the largest and smallest lesion size within a subject was 5189 (10046) mm^3^, demonstrating the large difference in lesion size within a patient. Table 2 demonstrates associations between the demographic/clinical predictors and T2 lesion number, total T2-LV and the maximum lesion size.

**Table 2:** Estimate and 95% confidence interval for the effect of demographic features on the T2 MRI outcomes

**Factor**	**Analysis**	Rate ratio for T2 lesion number*	Log-transformed T2 lesion volume**	Log-transformed maximum lesion volume**	**Log-transformed mean lesion size†**
Age	Unadjusted	1.00 (0.93, 1.07) P = 0.95	−0.10 (−0.22, 0.01) P = 0.08	−0.11 (−0.24, 0.01) P = 0.078	−0.02 (−0.06, 0.02) P = 0.266
	Adjusted	1.00 (0.93, 1.07) P = 0.99	−0.09 (−0.21, 0.03) P = 0.13	−0.10 (−0.23, 0.03) P = 0.12	−0.02 (−0.07, 0.02) P = 0.24
Gender (female/male)	Unadjusted	1.16 (0.75, 1.80) P = 0.51	−0.30 (−1.08, 0.48) P = 0.45	−0.35 (−1.20, 0.50) P = 0.41	−0.24 (−0.52, 0.03) P = 0.08
	Adjusted	1.24 (0.78, 1.96) P = 0.36	−0.22 (−1.08, 0.64) P = 0.61	−0.26 (−1.20, 0.67) P = 0.57	−0.26 (−0.55, 0.03) P = 0.07
Race (Non-white/White)	Unadjusted	1.20 (0.76, 1.87) P = 0.44	0.87 (0.10, 1.64) **P = 0.028**	1.16 (0.34, 1.99)^ **P = 0.0065**	0.01 (−0.27, 0.30) P = 0.92
	Adjusted	1.13 (0.69, 1.86) P = 0.62	0.87 (0.03, 1.72) **P = 0.044**	1.19 (0.29, 2.08)^ **P = 0.011**	0.01 (−0.30, 0.31) P = 0.97
Ethnicity (Hispanic/Non-Hispanic)	Unadjusted	1.39 (0.86, 2.22) P = 0.18	0.46 (−0.39, 1.32) P = 0.28	0.45 (−0.48, 1.39) P = 0.33	−0.14 (−0.43, 0.16) P = 0.36
	Adjusted	1.52 (0.86, 2.69) P = 0.15	0.36 (−0.68, 1.40) P = 0.49	0.25 (−0.89, 1.38) P = 0.66	−0.25 (−0.58, 0.09) P = 0.14
EDSS	Unadjusted	1.04 (0.86, 1.27) P = 0.67	0.20 (−0.15, 0.56) P = 0.25	0.22 (−0.16, 0.60) P = 0.26	0.05 (−0.06, 0.17) P = 0.36
	Adjusted	1.01 (0.82, 1.23) P = 0.95	0.17 (−0.21, 0.55) P = 0.36	0.19 (−0.22, 0.61) P = 0.35	0.07 (−0.05, 0.19) P = 0.25

Legend: *: negative binomial regression model, **: linear regression model, ^†^: repeated measures model, EDSS: expanded disability status scale, ^: p-value < 0.05. For negative binomial model, the rate ratio corresponds to a one-unit increase in age or EDSS and the difference between the groups for the other factors. For the linear regression model, the estimate is the increase in the log-transformed volume for a one-unit increase in age or EDSS and the difference between the groups for the other factors. Analyses were adjusted for disease duration and disease modifying therapy duration.

### Age

The mean age of subjects was 14.78 +/− 3.12 years reflecting the predilection for MS during the adolescent years. When analyzing MRI measurements, we observed no statistically significant associations between age and any of the T2 lesion measurements (Table 2) or number of GD + lesions (adjusted p = 0.84).

### Gender

Our model showed no statistically significant associations with any of the T2-LV or T2 lesion number measurements and gender. However, males in our model demonstrated a trend towards larger mean lesion size (adjusted p = 0.07) (Table 2). There was no statistically significant association between GD + lesion number and gender (adjusted p = 0.99).

### Race

We divided patients into groups of white and non-white patients, of which the latter contained both African-American patients and others (African-American = 8, Mixed origin and other = 8, American Indian = 1). We observed significant associations between race and log-transformed total T2-LV, as well as race and log-transformed maximum LV, from unadjusted (p_T2-LV_ = 0.028 and p_Max_ = 0.007) and adjusted analyses (adjusted p_T2-LV_ = 0.044 and adjusted p_Max_ = 0.011), with higher measures observed in non-white subjects in both cases. This association was not found using the log transformed mean T2 lesion size (adjusted p = 0.97). No significant differences were observed between white and non-white subjects in terms of number of T2 lesions (Table 2) or number of GD + lesions (adjusted p = 0.57).

### Ethnicity

There were no statistically significant associations with any of the T2 lesion measurements and ethnicity. No significant associations were observed between Hispanics and non-Hispanics in relation to number of GD + lesions (adjusted p = 0.42).

### EDSS

There was no significant association between EDSS score and any of the T2 MRI measures (see Table 2) or number of GD + lesions (adjusted p = 0.17).

## Discussion

Here, we study quantitative lesion characteristics in a multicenter cohort of children with MS. Our overall goals were to assess the characteristics of lesion volumetrics in this cohort and the feasibility of multicenter MRI studies in this population. Here, we demonstrate that multicenter quantitative MRI analysis is feasible among U.S. sites included in the Pediatric MS Network. We also provide cross-sectional data on a representative cohort of pediatric MS patients seen at these sites and associations with demographic features in this pilot study.

This is one of few studies using volumetric MRI analysis to assess children with MS, and adds to the limited knowledge in this patient population. 34% of patients had at least one gadolinium-enhancing lesion, suggesting ongoing disease activity in this “real world” cohort of pediatric MS patients. Our results show a wide range of both T2 (1–113) and GD + (0–32) lesion numbers in children with MS in the early phases of disease, demonstrating considerable heterogeneity amongst subjects.

We also explored potential correlates of this heterogeneity with demographic and clinical features. Our analysis found non-white patients to have a significantly higher T2–LV and maximum individual T2 lesion size than white patients. Although our model showed increased T2-LV and maximal T2-LV in non-whites, the mean T2 lesion size did not differ between whites and non-whites. This may be explained by the presence of more large as well as small lesions in the MRIs of non-whites. Prior results in adults have demonstrated that African-Americans have a more severe disease than white patients [15-17]. A small study found that pediatric MS patients of African-American descent have higher relapse rates than white children [18]. Our results needs to be replicated, and future studies should account for referral bias and include specific genetic markers of familial origin.

Longitudinal studies in adults have associated lesions larger than 600 mm^3^ with better recovery, whereas smaller lesions are associated with increased disability [19]. Whether this observation in adults applies to lesions in patients with pediatric MS warrants additional investigation in a longitudinal series.

Our results showed a trend towards larger mean T2 lesion size in boys than in girls. However, total T2-LV and number did not differ according to gender. In previous studies, pediatric MS patients under the age of 11 years had atypical MRI features with a higher frequency of confluent T2 lesions and fewer well-defined ovoid lesions than adolescents with MS [20]. We did not find an association between age and any of the volumetric measures studied, although qualitative lesion measures were not specifically examined.

In our cohort, a higher EDSS score was not significantly associated with any of the MRI measures assessed. This is not surprising given the overall low range of EDSS scores in our cohort, as well as prior studies in adult MS demonstrating poor correlation of this measure [21]. Cognitive deficits occur in 30-40% of children with MS [22,23]. Further studies correlating clinical and cognitive disability with MRI measures are required to provide insights into the substrate of deficits in pediatric MS.

Limitations of this study include the small number of subjects and potential referral bias. In addition, the use of different MRI scanners and slightly different MR pulse sequence parameters across sites are limitations in multicenter studies.

## Conclusions

This multicenter pilot MRI study has demonstrated associations of race with lesion measures. As our results are limited by small numbers and possible referral bias, this association needs to be validated in larger cohorts evaluating consecutive children presenting with MS. Additional studies are required to corroborate and further assess the underlying causes of these differences as well as longitudinal changes in MRI in children and adolescents with MS including response to specific treatments.

## Abbreviations

MRI: Magnetic resonance imaging; LV: Lesion volume; EDSS: Expanded disability status scale.

## Competing interests

The authors declare that they have no competing interests.

## Authors’ contributions

All authors contributed to the preparation of data and review of this manuscript. TC and RZ drafted the manuscript. All authors read and approved the final manuscript.

## Authors’ information

Statistical analysis was performed by Brian Healy (Biostatistics Center, Massachusetts General Hospital, Boston, MA).

## Pre-publication history

The pre-publication history for this paper can be accessed here:

http://www.biomedcentral.com/1471-2377/13/173/prepub
